# The School Clinic

**Published:** 1911-10-28

**Authors:** 


### THE SCHOOL CLINIC.

### II.—The General Practitioner's Point of View.

In principle no medical man objects to the medical inspection, supervision, and treatment of physically defective school children. As medical men we are well aware of the fact that such inspection is bound in the long run, if systematically and carefully carried out, to result in important data which may have a very extensive bearing upon our knowledge of children's diseases, their incidence, etiology, and pathology. The educative value of inspection is not yet fully realised by most of us, but once it is understood there can be no question that we shall all be whole-heartedly in favour of such systematic investigation. The results can never be final, but they will afford us a very valuable basis for classification, and will therefore be of the greatest use to the profession at large. But while we readily acknowledge the importance of the principle, we are by no means so willing or eager to subscribe cordially to its enforcement in practice. And let it be stated at the outset that this reluctance to put theories into practice is not due, as so many imagine it to be, to direct antagonism to the methods which have so far been used in the work. The main objection which the general practitioner holds is a relative rather than an absolute one. At present there is an unfortunate variance of method both in regard to inspection and in regard to treatment. It is much to be regretted that the local authorities do not see, or at all events do not appear to realise, the extent of this variation. Admittedly the personal equation in medical inspection, as The Hospital has pointed out in the preliminary article on general considerations, is very great. School doctors vary enormously in their capacity to disregard trivialities. What may be simply sufficient to warrant a note in the register, "This child suffers from adenoids and should have breathing exercises," to one inspector, may induce another inspector to write, "The child suffers from adenoids; a surgical operation is required." It is not necessary to lay stress on the equally great difference that exists between doctors in their capacity to judge the relative importance of heart murmurs and lung sounds, but every practitioner can furnish cases where a healthy child has been excused play or work on the score that the school doctor considers him to suffer from heart disease, or where the parents of a bronchiectatic child have been scared out of their wits by being told that the child is in an advanced stage of consumption. Our objection is that the local authorities do not make their appointments to the important posts of school doctors with sufficient discrimination. Few school doctors have had an extensive experience of diseases of children, and it is reasonable, in view of that fact, to ask local authorities to appoint as referees, who can investigate and in a consultative capacity report on doubtful cases, men who have an acknowledged eminence in pediatrics. Such an innovation will be productive of the greatest good to the children as well as to the system, for it will help to efface the unfortunate impression, which, rightly or wrongly, most practitioners have acquired, that the average school doctor exaggerates the importance of trivial defects.

When we come to the question of treatment, it is at once evident that there is in general a wide chasm of opinion between general practitioners and school doctors, or at least between such of the latter as are not general practitioners. The establishment of an independent school clinic in a poor-class neighbourhood—and it is mainly there that a school clinic is wanted—is indirectly a menace to the reputation and practice of every doctor in that district if it is staffed and managed by the local authorities from outside. It may be said that such an institution caters only for the poorer children whose parents cannot afford practitioners' fees, but a school clinic to be on a sound basis should demand a fee for every child treated, and there is no reason why this fee should be abstracted from the pockets of the already oversweated general practitioner. By sending the defective children to a hospital department it is argued that the best possible treatment is obtained, since they are seen and treated by recognised specialists. Anyone who has experience of the special departments in our large hospitals knows the fallacy of this. The work of treatment is in the majority of cases entrusted to a clinical assistant, who may even be an unqualified student. It is true the specialist supervises, but what, after all, does this supervision amount to? Most of us can give a long chapter of cases badly treated at hospitals, and in the circumstances it seems that the argument that superlatively good treatment is only possible at a hospital is entirely unfounded. Add to this that the taking of children by the parents for free treatment to the out-patient department of a general hospital is an abuse of hospital charity, and it is evident that the general practitioner has a strong case against the present scheme that is so popular with certain local authorities. The school clinic, on the other hand, if it is staffed by local practitioners who know the children and are acquainted with the conditions under which they live, may be productive of excellent results. Against such a "treatment centre" none of us object, but we hold that if we are to give our services at such a centre we should be adequately paid for them. We are quite capable of undertaking the main portion of the work that falls to such a clinic. Most of us, again, would approve of the attachment to each centre of a visiting medical or surgical referee who could act as a consultant in difficult cases: few of us, at any rate, will object to such an official. Under such an arrangement the interests of the children and of the practitioner would both be adequately safeguarded, much friction between school doctors and general practitioners would be obviated, and the results would be generally excellent. It is, at any rate, the ideal to be striven for, and general practitioners are by this time fully aware that it is a realisable ideal.

